# Animal health care seeking behavior of pets or livestock owners and knowledge and awareness on zoonoses in a university community

**DOI:** 10.14202/vetworld.2015.841-847

**Published:** 2015-07-10

**Authors:** Emmanuel J. Awosanya, H. O. Akande

**Affiliations:** Department of Veterinary Public Health and Preventive Medicine, Faculty of Veterinary Medicine, University of Ibadan, Ibadan, Oyo, Nigeria

**Keywords:** attitude, education, households, Nigeria, treatment, vaccination

## Abstract

**Aim:**

We investigated the attitude of pets or livestock owning households in a university community to animal health care services and assessed the knowledge and awareness level of the residents on zoonoses.

**Materials and Methods:**

Structured questionnaire was used to obtain information on demography, pet or livestock ownership, animal health care seeking behavior, awareness and knowledge of zoonoses from 246 households. We did descriptive statistics and bivariate analysis to determine the level of association in discrete variables between owners and non-owners of pets or livestock at a significant level of p<0.05.

**Results:**

Of the 246 respondents, 80 (32.5%) were either pet or livestock owners. The animal health care seeking behavior of the 80 pets or livestock owners in terms of treatment and vaccination was 70%. Of the 56 (70%) who provided health care services for their animals, about 48 (85.7%) engaged the services of a veterinarian. Dog owning households (42) had the highest frequency of treating their pets against endoparasites (97.6%); ectoparasites (81%) and vaccination against diseases (73.8%). Of the 246 respondents, only 47 (19.1%) have heard of the term zoonoses. Of the considered zoonoses; their awareness of rabies (79.3%) was the highest, followed by Lassa fever (66.3%), the least was pasteurellosis with 18.7%. Having pets or livestock was significantly associated (p=0.04) with rabies awareness. However, there is no significant difference in the level of awareness of zoonoses; knowledge of zoonoses, knowledge of prevention of zoonoses and knowledge of risk of zoonoses between owners and non-owners of pets or livestock.

**Conclusion:**

The animal health care seeking behavior of households with pets or livestock is good and should be encouraged. Public education should be created for other zoonoses aside from rabies, Lassa fever, and avian influenza.

## Introduction

Pets are companion animals, domesticated for pleasure rather than utility. They have psycho-social effects on their owners and are useful in the management of loneliness and stress [1]. Livestock such as pigs, goats, sheep, cow, and poultry are reared for food and other general purposes such as fertilizer and fuel, hides, and skins. It is the main stay of resource poor farmers who used livestock rearing as a means to an end [2]. Consequently, because of the major roles animals play in the life of humans; adequate health and care from qualified animal health providers should be made available to the pets and livestock; and veterinarians have been saddled with the role of providing information regarding prevention, control and notification of disease outbreaks; risk analysis, epidemiological surveillance and zoning; and administration of required treatments [3,4].

Zoonoses are diseases and infections that are naturally transmissible from vertebrate animals to humans and *vice versa* [5]. Although pets or livestock ownership is common in most countries; and non-owners of pets or livestock may have frequent contact with animals, there is limited knowledge of the public’s pet or livestock contact practices and awareness of zoonotic disease risks from pets or livestock [6].

The presence of roaming or stray animals in a university community, despite concerted efforts by the university management and the Veterinary Teaching Hospital (VTH) to rid the community of this menace has raised questions about the attitude or disposition of the residents to animal health care services. Risks for zoonoses are considered negligible compared with those for diseases of higher consequence because the societal consequences of zoonoses are not recognized by the individual sectors [7]. However, there is a need for improved education on zoonotic disease prevention practices for households with or without pets or livestock, particularly those with individuals at higher risk of infection [6].

Thus, this study aimed at investigating the attitude of pets or livestock owning households to animal health care services; and to assess the knowledge and awareness level of the residents on zoonoses. This will provide informed educational and communication intervention in zoonoses prevention and control to populations at risk and the entire university community.

## Materials and Methods

### Ethical approval

No ethical approval was necessary for this study; however, we obtained informed consent from all participants involved in the study and we maintained the confidentiality of the data obtained.

### Study design

This study was a cross-sectional survey among owners and non-owners of pets or livestock at the University of Ibadan, Ibadan, Nigeria from July to December 2013.

### Study sites

**Figure-1:**
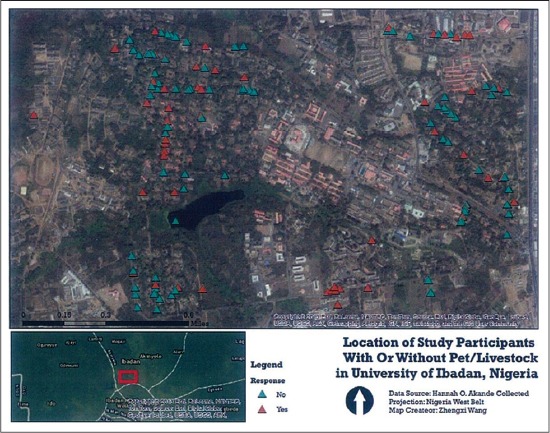
Location of households with or without pets or livestock at the University of Ibadan, Ibadan, Nigeria.

The study site was the University of Ibadan, Ibadan, which is on latitude 7°26’490” N and longitude 3°54’359” E (Geographical Positioning System, Etrex, Garmin, Taiwan). The University of Ibadan is the oldest University in Nigeria with a student population of about 20,000 [8]. The University has staff residential quarters that were used in this study (Figure-1). The University of Ibadan is a teaching and research institution. It has the presence of a VTH that is saddled with the responsibility of providing clinical training to veterinary students and offering of veterinary services, which range from diagnostic, therapeutic, surgical to preventive services, to the immediate community. It also handles referral clinical cases from state government and privately owned veterinary clinics.

### Study population

The respondents were residents at the University residential quarters who are owners and non-owners of pets or livestock. A respondent is anyone who is a member of a household selected in this study and who is responsible for the care of the pets or livestock in a household where they keep animals or who is old enough to speak for his or herself with minimal or no influence in a household where no animals are kept.

### Sample size and sampling

A total of 246 households were used for this study. We approached this by considering each of the residential quarters or streets as a sampling cluster. There were 38 clusters in all. We used the formula below c=p (p−1) D/s^2^b [9] to determine the number of clusters to be used for the study. Where p is the proportion of households with pets or livestock and assumed to be 50%; D is the design effect calculated to be 1.24 using the formula D=1+(b−1)*r. Where b is 13, which is the average number of household we want to sample in each cluster and r is the rate of homogeneity assumed to be 0.02. We wanted to achieve a precision of 5%, so the standard error (s) will be 0.025. This gave us 38.75~39 clusters. We adjusted for finite population correction using N/(n+N−1) and this gave us approximately 19 clusters. We sampled average of 13 households from each of 19 clusters selected from the list of 38 clusters using simple random sampling. However, we considered 10% non-response rate and administered a questionnaire to 271 households out of which 25 households were excluded.

### Data collection

We used a structured and pre-tested questionnaire to obtain information on the demography of respondents; pets or livestock ownership; animal health-seeking behavior; awareness and knowledge of zoonoses. The questionnaire was both interviewer and self-administered on request. We used hand held GPS (Etrex Garmin, Taiwan) device to take coordinates of sampled locations of participating households. The interviewers (3) were trained on how to administer the questionnaire in order to minimize interviewer bias. The questionnaire and its administration were done in English being the official language and the study site being a university community.

### Data analysis

The data were entered using Microsoft excel 2010. The data were cleaned and analyzed using EPI-Info version 3.5.4. Fifteen of the questionnaires were not returned, and we excluded 10 entries due to incomplete entries. This left us with a total of 246 households. We carried out descriptive statistics and bivariate analysis. We determined odds ratio and the statistical difference in discrete variables between owners and non-owners of pets or livestock using Chi-square test. Knowledge score on what zoonoses are and their prevention was put on a scale of 5; a score of equal or >3 and a score of equal or >2 is graded as good for what zoonoses are and knowledge on prevention respectively. Knowledge on the risk of zoonoses, pets or livestock could pose was scored using a 8-point knowledge scale; a score <4 was rated fair. A probability level of <5% (p<0.05) was accepted as significant.

## Results

### Demography

Of the 246 respondents, about half were males 124 (50.4%). The mean age was 29.9±12.1 years. Most of the respondents 131 (53.3%) are not married. The highest educational qualification of most was tertiary (those who had the post-secondary education or college degree) 199 (80.9%). About 120 (48.8%) were students; 111 (45.1%) were in the working class, the rest were unemployed. Of the 246 respondents, 80 (32.5%) households were either pet or livestock owners.

### Animal health care seeking behavior

**Figure-2:**
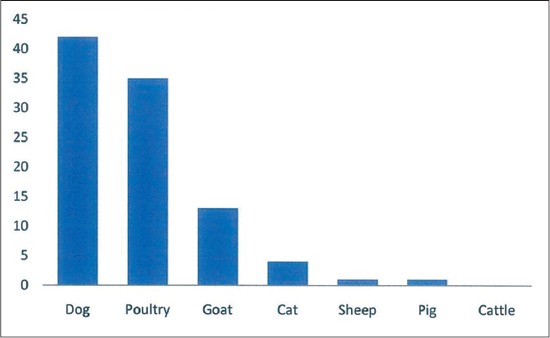
Dog is the most owned of the pet or livestock by the respondents.

**Figure-3:**
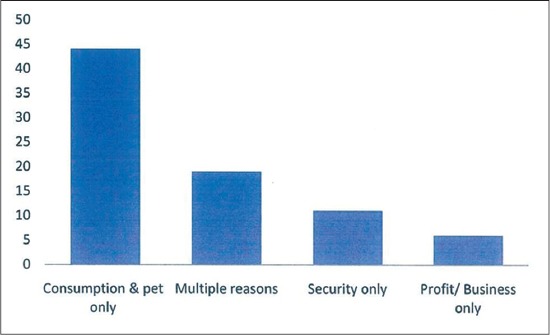
Most adduced reason for keeping animals by the respondents is for consumption or as pet.

**Figure-4:**
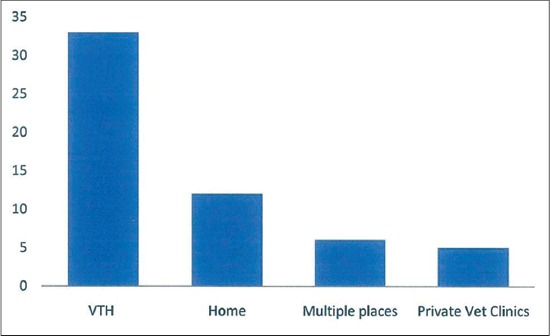
The Veterinary Teaching Hospital is the most sought place for animal health care services at the study site.

Of the 80 households who were either pet or livestock owners, only 41 (51.3%) kept their animals in strict confinement, 35 (43.7%) reared them semi-intensively while the rest allowed them to free range. Most of the respondents kept dogs 42 (52.5%); followed by poultry 35 (43.8%). None had cattle (Figure-2). Most adduced reason for keeping the animals was as a pet or for personal consumption 44 (50%). Only 6 (7.5%) reared them for profit making (Figure-3). Of the 80 pet or livestock owners, about 56 (70%) would treat or vaccinate their animals. Of the 56 (70%) who provided health care services for their animals, about 48 (85.7%) engaged the service of a veterinarian, only 6 (10.7%) give self-administered veterinary services. About 33 (58.9%) sought veterinary care services from the VTH (Figure-4). Of all the pet or livestock owners; pet owners (dogs 41 of 42 [97.6%] and cats 3 of 4 [75%]) are most likely to treat their pets against worms. Only 3 (23.1%) of the 13 goat owners and 6 (17.1%) of the 35 poultry owners reported treating against worms within the past 1-year, the rest livestock keepers had none. Pet owners (dogs 34 of 42 [81%] and cats 3 of 4 [75%]) are most likely to treat their pets against ectoparasites (ticks/lice/mites). Only 4 (11.4%) of the 35 poultry owners treated against ectoparasites, the rest livestock owners had no report within the past 1-year. Pet owners (dogs 31 of 42 [73.8%] and cats 1 of 4 [25%]) are most likely to vaccinate their pets against diseases within the past 1-year. Only 4 (11.4%) of the 35 poultry owners reported to have administered vaccination within the past 1-year. None of the other livestock owners reported to have given any form of vaccination within the past 1-year. Of the 56 health seeking animal owners, only 24 (42.9%) kept treatment or vaccination record. In general, of the 80 pet or livestock owners, 51 (63.8%) reported visiting a veterinary clinic at least once in the past 1-year; 44 (55%) reported calling a veterinary doctor at least once in the past 1-year; while 50 (62.5%) reported visiting human hospital at least once for treatment in the past 1-year.

### Knowledge and awareness on zoonoses

**Figure-5:**
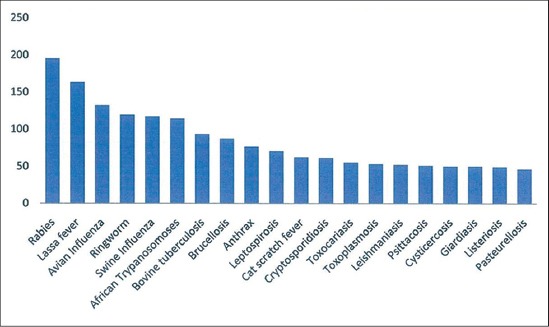
Rabies is the most heard of zoonoses by the respondents (n=246).

Of the 246 respondents, only 47 (19.1%) have heard of the term zoonoses. Most 22 (46.8%) of those who have heard of the term zoonoses attributed the source of their awareness to books and journals and 18 (38.3%) to the internet. Only 19 (40.4%) of the owners of pets or livestock have heard of the term zoonoses; there was no significant difference (p>0.05) in the awareness level among owners and non-owners of pets or livestock (Table-1). Of the 246 respondents, 41 (16.7%) of the respondents had good knowledge of what zoonoses are or could give at least three examples. Only 14 (34.1%) of owners of pets or livestock had good knowledge of what zoonoses are or could give at least three examples. However, there is no significant difference (p>0.05) among owners and non-owners of pets or livestock. Of 246 respondents, 30 (12.2%) had good knowledge of what to do to reduce incidence of zoonotic infection. Only 10 (33.3%) of the owners of pets of livestock had good knowledge of what to do to reduce incidence of zoonotic infection. There is no significant difference (p>0.05) in the knowledge of prevention between owners and non-owners of pets or livestock (Table-2). Of the considered zoonoses; their awareness of rabies (79.3%) was the highest, followed by Lassa fever (66.3%), the least was pasteurellosis with 18.7% (Figure-5). About 30.8% of the households owning pets or livestock were aware of rabies as a zoonosis. There is a significant difference (p<0.05) in the awareness level of rabies as a zoonosis among owners and non-owners of pets or livestock. Owners of pets or livestock are 2 times more likely to be aware of rabies as a zoonosis than non-owners of pets or livestock (Table-3). Of 246 respondents, 88 (35.8%) had good knowledge of the risk of zoonoses some pets and livestock poses to humans. Only 29 (33%) of the owners of pets or livestock had good knowledge of the risk of zoonoses pets or livestock could pose to humans; however, the difference was not significant between owners and non-owners of pets or livestock.

**Table-1:** Bivariate analysis of the awareness level on zoonoses among owners and non-owners of pet/livestock at the university community, 2013.

Variable	Have heard of the term zoonoses (%)	Have not heard of the term zoonoses (%)	OR	CI	p value
Owners of pets or livestock	19 (40.4)	61 (30.7)	1.5	0.80-2.96	0.27
Non-owners of pets or livestock	28 (59.6)	138 (69.3)			

OR=Odds ratio, CI=Confidence intervals

**Table-2:** Bivariate analysis of the knowledge of what to do to prevent incidence of zoonotic infection among owners and non-owners of pet/livestock at the university community, 2013.

Variable	Good knowledge of prevention (%)	Fair knowledge of prevention (%)	OR	CI	p value
Owners of pets or livestock	10 (33.3)	70 (32.4)	1.0	0.46-2.35	0.92
Non-owners of pets or livestock	20 (66.7)	146 (67.6)			

OR=Odds ratio, CI=Confidence intervals

**Table-3:** Bivariate analysis of the awareness of rabies as a zoonosis among owners and Non-owners of pet/livestock at the university community, 2013.

Variable	Aware of rabies as a zoonosis (%)	Not aware of rabies as a zoonosis (%)	OR	CI	p value
Owners of pets or livestock	70 (35.9)	10 (19.6)	2.3	1.1-4.9	0.04
Non-owners of pets or livestock	125 (64.1)	41 (80.4)			

OR=Odds ratio, CI=Confidence intervals

## Discussion

We reported a significant association between owning of pets or livestock and being aware of rabies as a zoonosis. This is similar to the observation of Awosanya and Adebimpe [10] who reported a similar association between rabies awareness and owning a dog in a university community in Nigeria. However, the study of Hergert and Nel [11] reported a higher awareness on rabies among non-owners than owners of dogs. The university community being a teaching and a research community and ease of access to veterinary care within the community could have positively influenced the behavior of the pet or livestock owning households towards search for relevant knowledge in the area of health care for their animals. Differences in the study communities, one being a university community and the other larger urban community may explain the dichotomy in the observation in awareness level from the two studies. In addition, the disparity in observation could be a reflection of the differences in intensity on public education on rabies in these communities.

This study revealed that household members in the study community had highest awareness level on rabies; followed by Lassa fever and avian influenza; which are all viral zoonoses. Awareness was lowest in pasteurellosis and listeriosis. A similar high awareness level for rabies was reported by Awosanya and Adebimpe [10], Hergert and Nel [11], and Tesfaye *et al*. [12]. The high rabies awareness could be adduced to the importance placed on rabies as a typical and zoonotic disease in Nigeria [13]. The awareness on Lassa fever is also high in this study; similar to the report of Tobin *et al*. [14]. The high awareness level on Lassa fever could be adduced to the enzootic status of Lassa fever in Nigeria [14,15] and the efforts of the Federal Ministry of Health together with her allies in public education to raise awareness and also keep the public informed on what to do should they notice any symptoms similar to the infection. The publicity given to avian influenza during its scourge in Nigeria [16] could be responsible for the high awareness level reported in this study. The low awareness level in some bacterial and parasitic zoonoses could be due to the low incidence and or the under-reporting of such cases. This trend is similar to the observation of Pfukenyi *et al*. [17] who reported high awareness of rabies as zoonoses and very low awareness of helminthes and protozoan parasites as zoonoses among pet owners in Zimbabwe.

We reported that most study participants had fair knowledge on what zoonoses are or their ability to mention at least three examples; what they can do to reduce incidence of zoonotic infection and what risk of zoonoses their pets or livestock could pose if neglected. Generally, there is no difference in the knowledge level between households that are owners and non-owners of pets or livestock. Previous studies have reported fair knowledge of study participants on the risk of zoonoses from their pets or having received information regarding pet-associated disease risks [6,17,18]. It appeared that the use of the term zoonoses for any disease or infection that are naturally transmissible from vertebrate animals to humans is limited to the veterinary world. Though some of the respondents could mention some infectious diseases that are zoonotic in nature but they could not relate to such diseases as zoonoses. It is believed that information, education and communication (IEC) materials should be suitable, of simple language and having effective message. However, the problem of inability to relate diseases that are transmissible from animals to humans and *vice versa* to zoonoses should be addressed by IEC materials developed by local, state and national government for each zoonosis. For instance, a member of a society should be able to recognize rabies as a zoonosis. Veterinary extension is also of utmost importance in addressing some of the deficiency in knowledge by most of the respondents. Public health veterinarians should see it as part of their roles to educate their clients on the risk of zoonoses from pets and or livestock [3]. A study by Pfukenyi *et al*. [17] reported that more than 50% of pet owners indicated that their veterinarians never discussed the potential hazards of zoonoses.

Among households that are owners of pets or livestock, a good number would treat and or vaccinate their animals; a very high percentage would request the service of a veterinarian or visit the VTH. The positive attitude of the households who were owners of pets or livestock towards seeking veterinary services could be attributed to the presence and nearness of VTH in the community. Presence and closeness of veterinary health centers in a community have been reported to influence access to veterinary services and choice of veterinary service providers [19-21]. Most of the respondents had a tertiary education (i.e., had the post-secondary education or a college degree); this also could have influenced their positive attitude towards seeking veterinary services from a veterinarian. Beam *et al*. [20] reported that small-scale producers with college degrees were significantly more likely to use a veterinarian than those who are not. Just about half of the households that are owners of pets or livestock in the study area keep them in strict confinement. It could be explained from this perspective that most of the roaming animals in the study area are owned and might have received some level of veterinary care services.

This study revealed that households that are pet (dog and cat) owners are more likely to treat their pets against worms than households that are livestock owners (poultry, sheep, goats, and pigs). More so, households with pets are more likely to treat their pets against ectoparasites that livestock owning households. The same trend is observed for vaccination. The reason could be that most households that are owners of dogs and cats keep them for companion; while most households that are owners of livestock keep them for the purpose of household consumption and may not want to incur much expense on providing veterinary services. More so, dog rabies vaccination is done to prevent rabies cases in dogs and by extension to minimize human cases of rabies through the bite of an infected dog. The VTH annually commemorates the World Rabies Day every 28^th^ September during which anti-rabies campaign is intensified and occasionally free rabies vaccination is given. The high awareness level on rabies [10,12] could have positively impacted the health seeking behavior of the households that are owners of dogs or cats towards deworming, ectoparasites control and vaccination of their pets.

Keeping of health records enhances better communication between the clients and their veterinarians, disease monitoring and control. We reported a poor health record keeping attitude among those households who reported treating or vaccinating their animals. This could be because very few of them keep the animals for profit or commercial purposes. It could also be due to ignorance on the benefits of keeping such records. This is similar to the observation of Awosanya and Adebimpe [10] who reported under-reporting of dog bite cases in a university community.

This study may be limited by both information and interviewer biases as they are common with questionnaire administration. We reduced the effect of information bias in the design of the questionnaire; we asked certain questions that require consistency with answers to previously asked questions. For instance, when we ask who is responsible for the vaccination of your dog and the response is a veterinarian; down the line of the questionnaire we ask again how many times in the past 1-year have you called or visited a veterinarian? We mitigated interviewer bias by the training of the interviewers used in this study in order to ensure uniformity in both spoken and body language. These limitations were taken into consideration in the interpretation of the data.

## Conclusion

The animal health care seeking behavior of households with pets or livestock is good and should be further encouraged. Most of the pet or livestock owners sought animal health care from qualified veterinarians and at the VTH. Pet owners are more likely to seek animal health care than their livestock counterpart. Animal health care seeking behavior should be encouraged among livestock owners. There is poor health record keeping among pets or livestock owners, health record keeping should be encouraged. There is also a general poor knowledge on common zoonoses. Public awareness on Rabies, Lassa fever, and avian influenza is above average while others are below. Public awareness should be created for other zoonoses aside from rabies, Lassa fever and avian influenza especially at the VTH and veterinary clinics to owners of pets or livestock who are a set of population at risk group.

## Authors’ Contributions

EJA conceived and designed the study. HOA acquired the data. Both authors were involved in the analysis, interpretation, drafting, and revising of the manuscript. Both authors read and approved the final version of the manuscript.
